# Effects of thickness and polishing treatment on the translucency and opalescence of six dental CAD-CAM monolithic restorative materials: an in vitro study

**DOI:** 10.1186/s12903-023-03299-y

**Published:** 2023-08-19

**Authors:** Zhengda Wu, Jiehua Tian, Donghao Wei, Yifan Zhang, Ye Lin, Ping Di

**Affiliations:** Department of Implantology, Peking University School and Hospital of Stomatology, 22 South Zhongguancun Avenue, Haidian District, Beijing, 100081 China

**Keywords:** CAD-CAM Materials, Translucency, Opalescence, Thickness, Roughening

## Abstract

**Background:**

Computer-aided design and computer-aided manufacturing (CAD-CAM) materials for prosthetic is gaining popularity in dentistry. However, limited information exists regarding the impact of thickness and roughening treatment on the optical properties of contemporary CAD-CAM restorative materials. This study aimed to quantitatively evaluate the translucency and opalescence of six dental CAD-CAM materials in response to different thicknesses and roughening treatments.

**Methods:**

Six dental CAD-CAM materials, lithium disilicate glass–ceramic (IPS e.max CAD, LS), polymer-infiltrated ceramic (VITA Enamic, VE), resin-nano ceramic glass–ceramic (LAVA Ultimate, LU), polymethyl methacrylate (Telio CAD, TE), and two zirconia reinforced lithium silicate (VITA Suprinity, VS, and Celtra Duo, CD), in shade A2 were prepared as 12 × 12mm^2^ specimens of four thicknesses (0.5mm, 1.0mm, 1.5mm, and 2.0mm) (*N* = 240, *n* = 10). After three different treatments (polished, roughened by SiC P800-grit, and SiC P300-grit), the translucency parameter (TP_00_) and opalescence parameter (OP) were measured with a spectrophotometer (VITA Easyshade V). The surface roughness was analyzed with a shape measurement laser microscope. The data were analyzed using a MANOVA, post hoc Tukey–Kramer test, the *t* test, and regression analysis (α = .05).

**Results:**

The TP_00_ and OP were significantly influenced by material type, thickness and roughening treatment (*P* < .05). TP_00_ showed a continues decline with increasing thicknesses, while the variations of OP were material-dependent. TP_00_ ranged from 37.80 (LS in 0.5mm) to 5.66 (VS in 2.0mm), and OP ranged from 5.66 (LU in 0.5mm) to 9.55 (VS in 0.5mm). The variations in TP_00_ of all materials between adjacent thicknesses ranged from 2.10 to 15.29, exceeding the acceptable translucency threshold except for LU. Quadratic and logarithmic regression curves exhibited the best fit for TP_00_ among the materials. Compared to polished specimens, rougher specimens exhibited lower TP00 and higher OP in all materials except for LS (*P* < 0.05). Roughening with P300-grit decreased TP_00_ and OP by an average of 2.59 and 0.43 for 0.5mm specimens, and 1.26 and 0.25 for 2.0mm specimens, respectively.

**Conclusions:**

Variations in translucency caused by thickness and roughening treatment were perceptible and may be clinically unacceptable. Careful consideration should be given to the selection of CAD-CAM materials based on their distinct optical properties.

## Background

Dental computer-aided design and computer-aided manufacturing (CAD-CAM) restorative materials have gained popularity in dentistry for indirect restorations [1]. The optical properties of CAD-CAM materials play a crucial role in restorative dentistry, aiming to recreate natural dental structures from esthetic perspective. To achieve excellent esthetics, it’s essential for the restorative team to possess a thorough understanding of the basic principles and optical characteristics of CAD-CAM materials to replicate the complex optical appearance of affected teeth [2].

Translucency and opalescence are key factors in achieving natural-looking results, which should dental restorations exhibit comparable to adjacent teeth [3, 4]. Translucency refers to the amount of light transmitted or diffused from the substrate, representing the material's state between complete opacity and transparency [3, 5]. The translucency parameter (TP) is commonly used in esthetic dentistry and calculated as the color difference from a white and black background using the Commission Internationale de l’Éclairage (CIE) color space, allowing quantitative evaluation of translucency [6]. Higher TP values indicate higher translucency. Opalescence is the optical characteristic of dental materials that exhibit a bluish-white appearance in reflected light and an orange-brown appearance in transmitted light, which is evaluated as opalescence parameter (OP) [3, 7]. This characteristic arises from the light scattering phenomenon caused by shorter or equal wavelengths of the visible spectrum in translucent materials [3, 8]. The opalescence of materials contributes to the masking of background color along with translucency, particularly when translucency is within a similar range [9].

The translucency and opalescence of CAD-CAM restorations, utilizing monolithic blocks, can be influenced by various factors, including material type, thickness, and surface treatments [3, 8, 10–16]. Firstly, fabricating esthetic dental restorations poses significant challenges for dental technicians due to the varying thickness requirements for each restoration, greatly impacting translucency and opalescence. The esthetic success of tooth-colored restorations often relies on the experience and skill of laboratory technicians in handling translucent materials [17]. As translucency and opalescence prediction is a rapidly growing research area in dentistry [16, 17], comprehensive knowledge of expected changes in translucency and opalescence based on material thickness is crucial for successful dental restorations. Several studies have reported a correlation between translucency and thickness, demonstrating a decrease in translucency values with increasing thickness [8, 12–16]. However, a precise mathematical formula for this correlation remains elusive due to significant variations among different studies [16, 17]. Consequently, obtaining color information at different thicknesses and accurately understanding the quantitative relationship are essential initial steps towards achieving predictable and highly aesthetic CAD-CAM restorations [8, 16, 17].

Meanwhile, there is a need for quantitative studies to determine whether variations in translucency are perceptible or clinically acceptable. Errors in translucency are particularly noticeable as they are closely tied to the lightness of a material, and the human eye is more sensitive to differences in lightness than hue or chroma [18]. Visual translucency thresholds have been widely employed as quality control tools and guides for evaluating translucency differences in dental materials, as well as in the analysis of clinical and in vitro research findings [6, 19]. Translucency thresholds for restorative dental materials using TP_00_ have been studied by Salas et al. [6], who assessed the basis of 50:50% translucency acceptability thresholds at 2.62 units and perceptibility thresholds at 0.62 units.

Secondly, the optical properties of CAD-CAM materials may undergo changes during prosthesis repair or adjustments such as grinding or polishing [20–22]. Meanwhile, wear, aging, and acid etching occur naturally to the restoration [23–25]. These factors could alter the topography and roughness of CAD-CAM materials, consequently influencing light transmittance and altering translucency and opalescence [23–30]. Previous studies have primarily focused on comparing translucency and opalescence results between different surface treatments, such as glazing or aging. However, there is a need to quantitatively evaluate the degree of color change after multiple roughening treatments to simulate the daily wear of CAD-CAM restorations.

Thirdly, various materials for CAD-CAM restorations, including glass–ceramics, zirconia, and composites, are available in dentistry currently [31]. Although manufacturers claim good translucency for these CAD-CAM materials, independent data comparing the materials on the market are limited. The quantitative relationship between translucency, opalescence, and thickness, as well as the differences in translucency and opalescence among different CAD-CAM materials, remains unclear, posing challenges in material selection and replicating tooth color.

Therefore, the aim of this study was to quantitatively evaluate and compare differences in translucency and opalescence among six different contemporary CAD-CAM materials, considering clinically relevant thicknesses and roughening treatments. The null hypothesis posited that material type, material thickness, and roughening treatment would not affect translucency and opalescence.

## Material and methods

### Specimens preparation

The six dental CAD-CAM restorative materials tested in this study are outlined in Table 1. The sample size was determined based on the findings of previous studies [3, 12, 14, 32]. Using power analysis software PASS 2021 (NCSS, LLC. Kaysville, Utah, USA), a minimum of 8 specimens for each material and thickness was calculated to achieve 80% power (β = 0.2), a two-sided statistical significance level of 5% (α = 0.05), and a detectable difference of 0.1. As a result, a total of 240 specimens measuring 12 × 12 mm in shade A2 were fabricated, with 10 specimens prepared for each material and four thicknesses (0.5mm, 1.0mm, 1.5mm, and 2.0mm) [12]. The specimens were obtained using a precision wire cutting machine (STX-2-2A; Shenyang Kejing Automation Equipment Co Ltd., Shenyang, China) operating at a low speed of 0.2mm/min and constant water cooling [32]. For VITA Suprinity blocks (VITA Zahnfabrik, Bad Säckingen, Germany) and IPS e.max CAD blocks (Ivoclar Vivadent AG, Schaan, Liechtenstein), the specimens were subsequently sintered in a ceramic furnace (Programat EP 5000; Ivoclar AG, Schaan, Liechtenstein) following the manufacturer's specifications [12, 32].

**Table 1:** Details and codes of tested materials

Material	Brand	Code	Main components^a^	Manufacturer
Lithium-disilicate ceramic	IPS e.max CAD	LS	8–80% SiO_2_, 11–19% Li_2_O, 0–13% K_2_O, 0–8% ZrO_2_, 0–5% Al_2_O_3_	Ivoclar AG, Schaan, Liechtenstein
Polymer-infiltrated ceramic	Vita Enamic	VE	86% ceramic (58–63% SiO_2_, 20–23% Al_2_O_3_, 9–11% Na_2_O, 4–6% K_2_O, 0–1% ZrO_2_) 14% polymer (UDMA, TEGDMA)	VITA Zahnfabrik, Bad Säckingen, Germany
Resin nanoceramic	Lava Ultimate	LU	80% ceramic (69% SiO_2_, 31% ZrO_2_) 20% polymer (UDMA)	3M ESPE, St. Paul, MN, USA
Polymethyl methacrylate (PMMA)	Telio CAD	TE	99.5% PMMA polymer	Ivoclar AG, Schaan, Liechtenstein
Zirconia-reinforced lithium silicate ceramic	VITA Suprinity	VS	56–64% SiO_2_, 1–4% Al_2_0_3_, 15–21% Li_2_O, 8–12% ZrO_2_, 1–4% K_2_O	VITA Zahnfabrik, Bad Säckingen, Germany
Zirconia- Reinforced Lithium Silicate ceramic	Celtra Duo	CD	58% SiO_2_, 18.5% Li_2_O, 5% P_2_O_5_, 10.1% ZrO_2,_ 1.9% Al_2_O_3_, 2% CeO_2_, 1% Tb_4_O_7_	Dentsply Sirona, Charlotte, USA

*TEGDMA* Triethylene glycol dimethacrylate, *UDMA* Urethane dimethacrylate

^a^As reported by manufacturers

To achieve uniformity, all specimens underwent sequential polishing on both sides using wet silicon carbide paper (Suisun Co Ltd., Hong Kong, China) until SiC P2000-grit on a grinding machine (M-Prep; Allied High Tech Products Inc., Rancho Dominguez, CA, USA) [12]. Subsequently, surface roughening treatments were applied to one side of the specimens using wet silicon carbide paper (Suisun Co Ltd., Hong Kong, China) at SiC P300-grit and SiC P800-grit (M-Prep; Allied High Tech Products Inc., Rancho Dominguez, CA, USA), performed by the same experienced operator (W.Z) [12, 32]. The operator was well-trained and demonstrated good intra-operator reliability in performing surface roughening treatments. Specimen thicknesses were determined using a digital micrometer with an accuracy of 0.02mm (Mitutoyo IP65, Mitutoyo Corp., Tokyo, Japan) [12, 32]. Prior to translucency and opalescence measurements, all specimens underwent ultrasonic cleaning in distilled water for 10 min, followed by cleaning with isopropanol to remove grease residue and drying with compressed air [8].

### Translucency and opalescence measurements

The CIELab coordinates (L*, a*, b*, C* and H*, which represent lightness, the red-green axis, the yellow-blue axis, chroma and hue, respectively) of each specimen were obtained using a dental spectrophotometer (VITA Easyshade V; VITA Zahnfabrik, Bad Säckingen, Germany) in “tooth single” mode under D65 illumination. The spectrophotometer employed an integrated illumination with a built-in white LED light source (D65) with 2-degree standard observer and (45:0) optical geometry [33], which could obtain CIE L*a*b* parameters with a repeatability less than 0.1 units and represent high inter-device and intra-device reliability [34]. Measurements were taken on a standard white background (L* = 99.0, a* = 0.0, b* = 2.2, C = 2.2, H = 90) and black background (L* = 1.15, a* = 0.3, b* = -2.0, C = 0.4, H = 326.2). The Ø5-mm probe was placed at the center of the specimen surface, and measurements were taken by the same experienced operator (W.Z). Prior to each measurement, the spectrophotometer was calibrated following the manufacturer's guidelines. The operator was well-trained and exhibited good intra-operator reliability in performing the measurements. Three sets of measurements were obtained, and the order of measurement for each group was randomized using the random number table method in each set. The mean values of the three measurements were then calculated for each specimen.

Translucency was evaluated by calculating the CIEDE2000 translucency parameter (TP_00_) based on the differentiation of coordinates measured on the black and white backgrounds using the CIEDE2000 (1:1:1) color difference formula [6]:

$$TP^{00}=\sqrt{{(\frac{{L}_{B}^{\mathrm{^{\prime}}}-{L}_{W}^{\mathrm{^{\prime}}}}{{K}_{L}{S}_{L}})}^{2}+{(\frac{{C}_{B}^{\mathrm{^{\prime}}}-{C}_{W}^{\mathrm{^{\prime}}}}{{K}_{C}{S}_{C}})}^{2}+{(\frac{{H}_{B}^{\mathrm{^{\prime}}}-{H}_{W}^{\mathrm{^{\prime}}}}{{K}_{H}{S}_{H}})}^{2}+{R}_{T}(\frac{{C}_{B}^{\mathrm{^{\prime}}}-{C}_{W}^{\mathrm{^{\prime}}}}{{K}_{C}{S}_{C}})(\frac{{H}_{B}^{\mathrm{^{\prime}}}-{H}_{W}^{\mathrm{^{\prime}}}}{{K}_{H}{S}_{H}})}$$

where the subscript B represents a black background, and the subscript W represents a white background. The parametric factors K_L_, K_C_, K_H_, S_L_, S_C_, S_H_ and R_T_ were set to 1, as previously described [6]. A CIEDE2000 50:50% translucency perceptibility threshold (TPT) of 0.62 units and acceptability threshold (TAT) of 2.62 units by Salas et al. were utilized [6].

Opalescence was evaluated by calculating the opalescence parameter (OP) based on the differentiation of blue-yellow and green–red coordinates using the following formula [7]:

$$OP=\sqrt{{({a}_{B}^{*}-{a}_{w}^{*})}^{2}+{({b}_{B}^{*}-{b}_{w}^{*})}^{2},}$$

where the subscript B represents a black background, and the subscript W represents a white background.

### Roughness measurements

The specimens were analyzed with a shape measurement laser microscope (VK-X200, Keyence, Osaka, Japan). The probe of the laser microscope was positioned at the center of the specimen surface, and three sets of measurements were taken for each group using the random number table method to obtain an average roughness profile.

### Statistical analysis

Statistical analyses were conducted by an experienced statistician (T.J), who was blinded to sample preparation and measurements, using a software program (IBM SPSS Statistics, v25.0; IBM Corp., Armonk, NY, USA) (α = 0.05). Results of the Shapiro–Wilk test and Levene test determined that the data were normally distributed and homogeneous (*P* > 0.05). The influence of material type, thickness, and roughening treatment on translucency and opalescence were analyzed by using a MANOVA (α = 0.05). Pairwise comparisons between the tested groups were performed using the post hoc Tukey–Kramer test (α = 0.05). The translucency and opalescence difference compared with the perceptibility and acceptability thresholds were analyzed using the *t*-test. To analyze the relationship between TP_00_ and thicknesses of the tested materials, four regression analyses (linear, exponential, logarithmic, and quadratic) were employed.

## Results

Table 2 summarize the results of MANOVA on the effects of material type, thickness, and roughening treatment on TP_00_ and OP. The analysis revealed significant influences of material type, thickness, and roughening treatment on both translucency and opalescence (*P* < 0.05).

**Table 2:** Summary of MANOVA results of TP_00_ and OP

Value	Source of variation	Type III Sum of Squares	Df	Mean Square	F	η_P_^2^	*P*
TP_00_	Type	2207.223	5	441.445	20.158	.307	< .001
	Thickness	7289.276	3	2429.759	110.951	.595	< .001
	Roughening	144.247	2	72.124	3.293	.028	.039
	Type * Thickness	515.289	15	34.353	1.569	.094	.084
	Type * Roughening	281.876	10	28.188	1.287	.054	0.239
	Thickness * Roughening	90.943	4	22.736	1.038	.018	.388
	Type * Thickness * Roughening	470.455	20	23.523	1.074	.086	.378
	Error	4971.162	227	21.899	-	-	-
OP	Type	743.683	5	148.737	1014.835	.957	< .001
	Thickness	37.898	3	12.633	86.194	.533	< .001
	Roughening	2.618	2	1.309	8.931	.073	< .001
	Type * Thickness	489.961	15	32.664	222.868	.936	< .001
	Type * Roughening	13.730	10	1.373	9.368	.292	< .001
	Thickness * Roughening	3.373	4	.843	5.753	.092	< .001
	Type * Thickness * Roughening	9.903	20	.495	3.378	.229	< .001
	Error	33.270	227	.147			

**Fig. 1:**
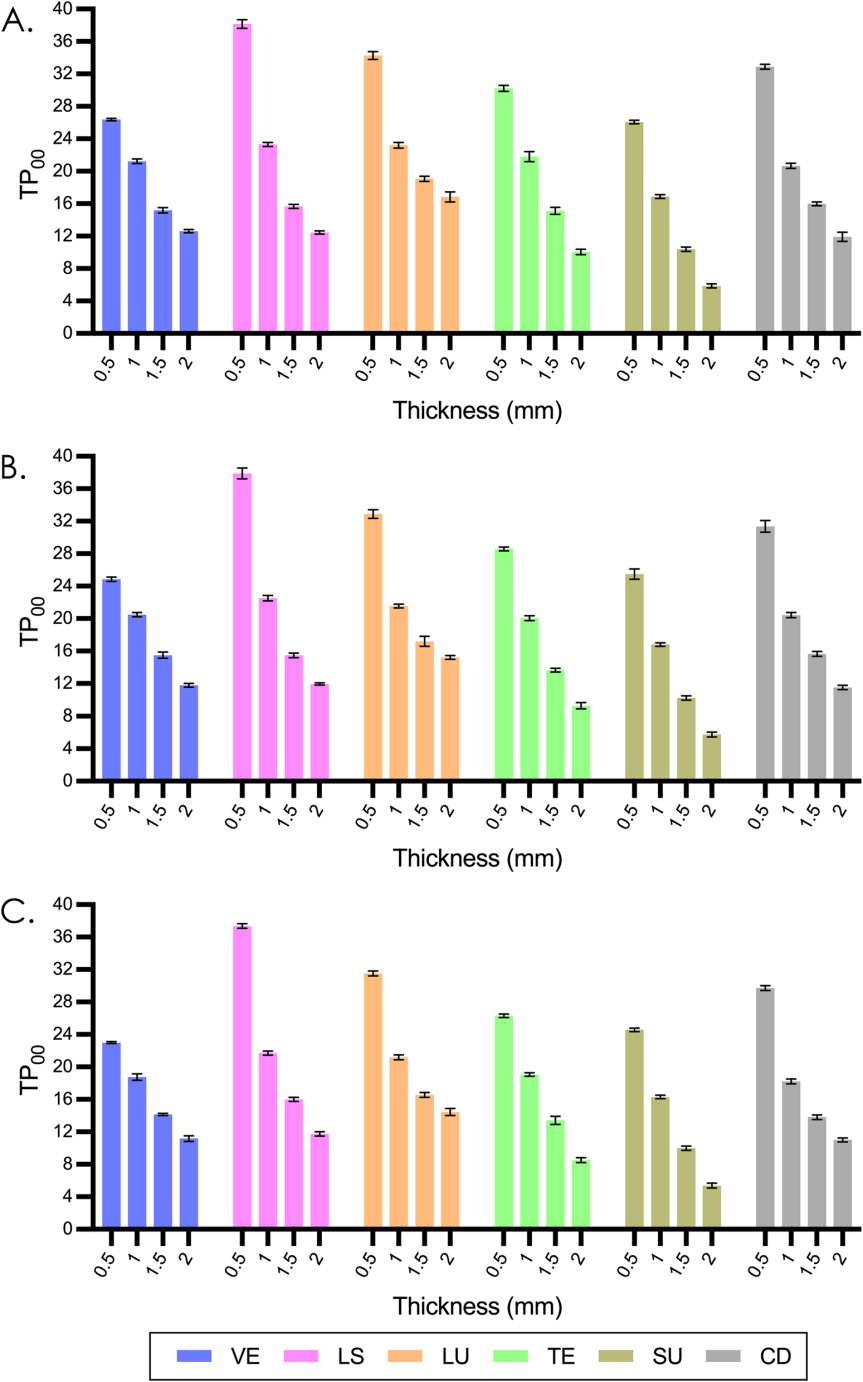
Mean and standard deviation results of TP_00_ in different thicknesses. **A**, Polished groups. **B**, SiC P800-grit roughened groups. **C**, SiC P300-grit roughened groups. VE, VITA Enamic; LS, IPS e.max CAD; LU, LAVA Ultimate; TE, Telio CAD; VS, VITA Suprinity; CD, Celtra Duo

**Fig. 2:**
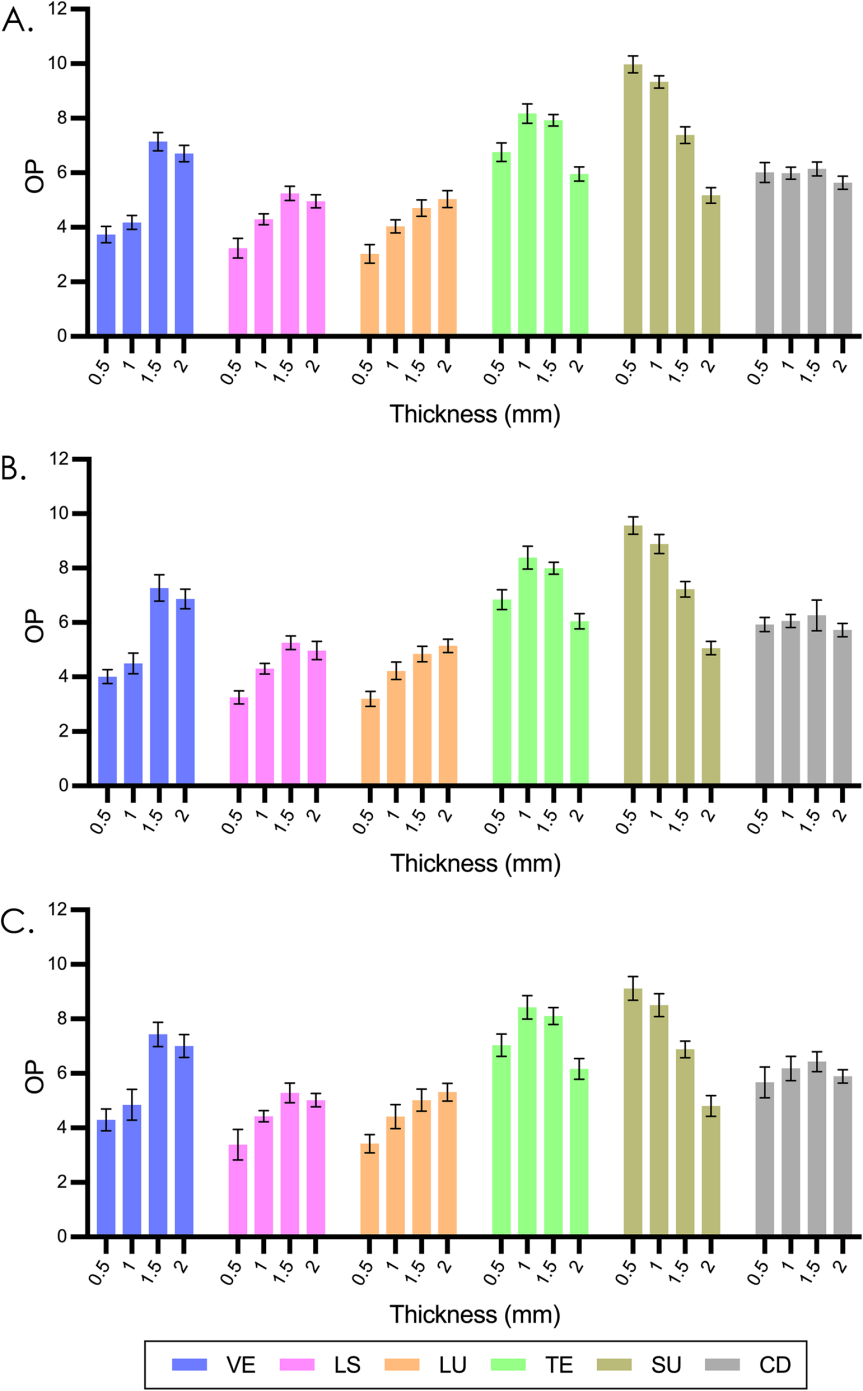
Mean and standard deviation results of OP in different thicknesses. **A**, Polished groups. **B**, SiC P800-grit roughened groups. **C**, SiC P300-grit roughened groups. VE, VITA Enamic; LS, IPS e.max CAD; LU, LAVA Ultimate; TE, Telio CAD; VS, VITA Suprinity; CD, Celtra Duo

Figures 1 and 2 display the mean and standard deviation values of TP_00_ and OP. A general decrease in TP_00_ (average from 30.08 to 10.97) was observed as the thickness increased. TP_00_ ranged from 37.80 (observed in 0.5mm LS) to 5.66 (observed in 2.0mm VS). The OP of most materials increased firstly and then decreased with increasing thickness, with the exception of LU showed continuous increase and VS showed continuous decrease. OP ranged from 5.66 (observed in 0.5mm LU) to 9.55 (observed in 0.5mm VS).

**Fig. 3:**
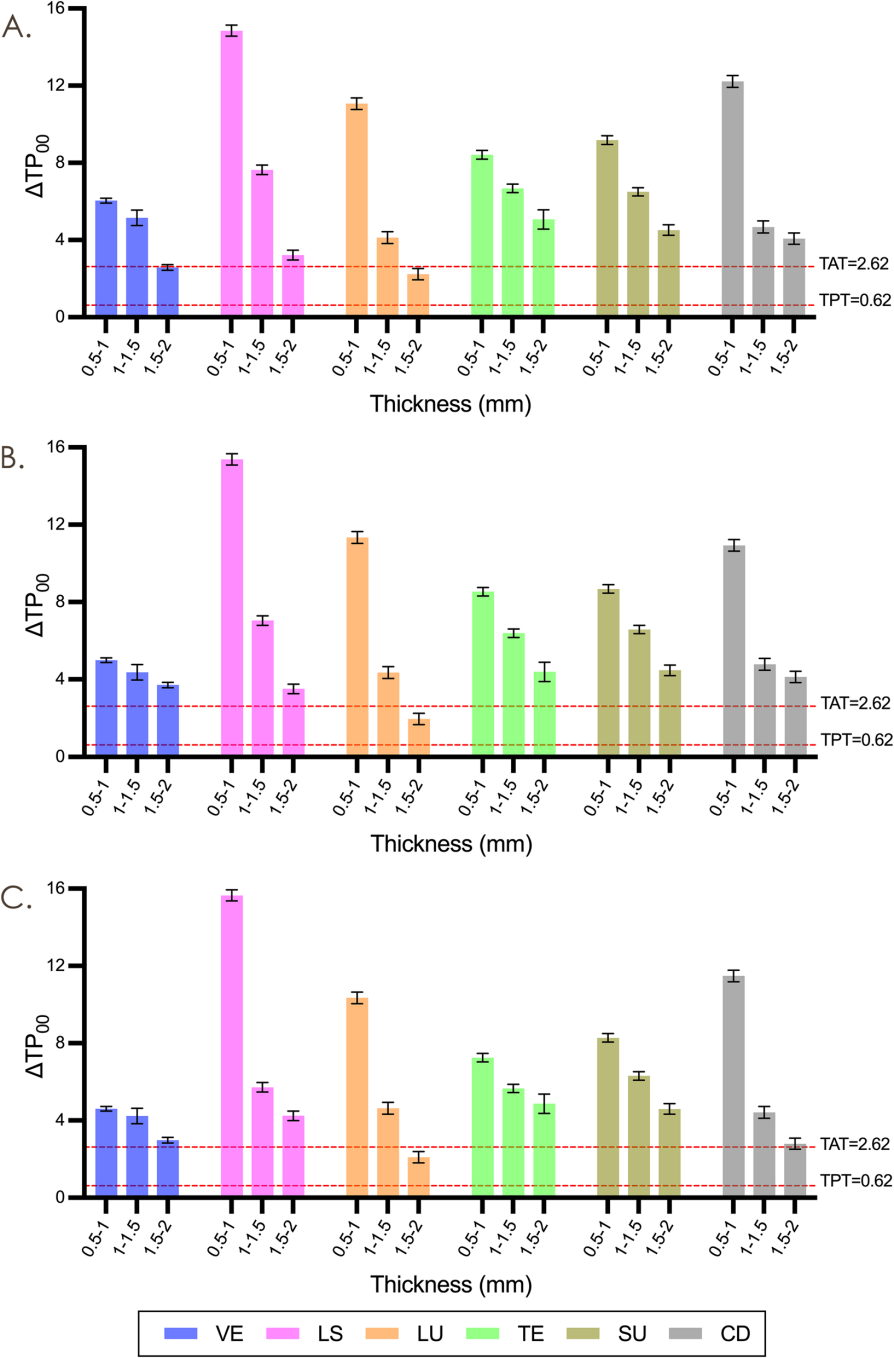
Mean and standard deviation results of TP_00_ between adjacent thicknesses. **A**, Polished groups. **B**, SiC P800-grit roughened groups. **C**, SiC P300-grit roughened groups. VE, VITA Enamic; LS, IPS e.max CAD; LU, LAVA Ultimate; TE, Telio CAD; VS, VITA Suprinity; CD, Celtra Duo

The variations in TP_00_ (ΔTP_00_) between adjacent thicknesses for the same material (Fig. 3) showed a decline as the thickness increased, ranging from 9.85 (between 0.5mm and 1.0mm) to 3.64 (between 1.5mm and 2.0mm). The highest variations in TP_00_ were observed in LU between 1.5mm and 2.0mm (ΔTP_00_ = 2.10) and lowest were observed in LS between 0.5mm and 1.0mm (ΔTP_00_ = 15.29). All variations were higher than the TAT, except for LU between 1.5 and 2.0mm. The variations in OP ranged from 0.20 (CD between 0.5mm and 1.0mm) to 2.77 (VE between 1.0mm and 1.5mm).

**Fig. 4:**
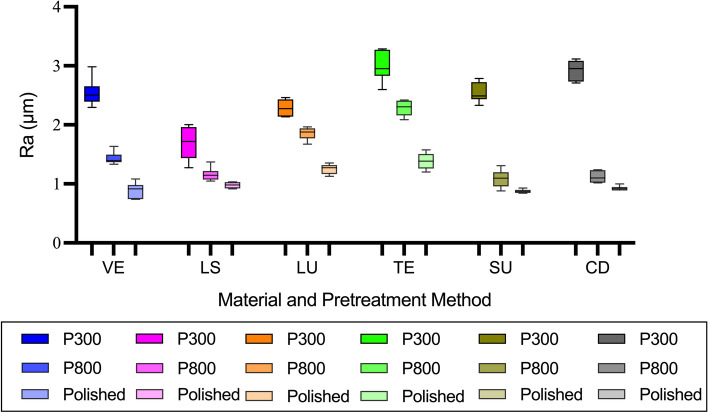
Mean and standard deviation results of surface roughness after different treatment methods. VE, VITA Enamic; LS, IPS e.max CAD; LU, LAVA Ultimate; TE, Telio CAD; VS, VITA Suprinity; CD, Celtra Duo

Significant correlations between TP_00_, OP, and roughening treatments were observed in all materials except for LS (*P* < 0.05). Figure 4 illustrates the surface roughness of materials after different treatments. Rougher specimens exhibited lower TP_00_ and higher OP (*P* < 0.001). Roughening by P300-grit decreased TP_00_ and OP by an average of 2.59 (close to TAT) and 0.43 for 0.5mm specimens, while 1.26 (higher than TPT but lower than TAT) and 0.25 for 2.0mm specimens compared to the polished ones. The variations in TP_00_ between roughening treatments ranged from 0.21 (2.0mm LS between P800-grit and P300-grit roughened) to 3.91 (0.5mm TE between polished and P300-grit roughened), while the variations in OP between roughening treatments ranged from 0.03 (2.0mm LS between P300-grit and P800-grit roughened) to 0.85 (0.5mm VS between polished and P300-grit roughened).

The analysis of the regression curves (linear, exponential, logarithmic, and quadratic) for the tested materials indicated that the quadratic regression curves provided the best fit (R^2^ closer to 1.0) for VE, LU, TE, and SU, while logarithmic regression curves provided the best fit for LS and CD (Table 3).

**Table 3:** R^2^ values of linear, exponential, logarithmic, and quadratic curves of all materials

Material	Linear	Exponential	Logarithmic	Quadratic
VE	.965	.966	.957	.976
LS	.953	.944	.997	.996
LU	.963	.978	.983	.984
TE	.986	.985	.980	.993
SU	.976	.982	.994	.997
CD	.918	.975	.987	.985

## Discussion

The results of this study rejected the null hypothesis, indicating that material type, thickness, and roughening treatment all had significant effects on translucency and opalescence.

Translucency and opalescence of dental materials are essential factors in achieving natural-looking dental restorations [3]. Dentists and technicians commonly evaluate these characteristics visually or using digital techniques. However, visual assessment is subjective and can be influenced by external factors such as ambient light and individual observers [35, 36]. To obtain a more objective analysis, spectrophotometers, like the Vita Easyshade V used in this study, offer clinically accurate and acceptable measurements of translucency and opalescence [34, 37].

Accurately predicting translucency and opalescence that closely resemble natural teeth in CAD-CAM restorations remains a challenge. The aesthetic success of prostheses often relies on the expertise of laboratory technicians working with translucent materials. As the prediction of translucency and opalescence continues to advance, gaining precise knowledge of how these characteristics change with material thickness based on mathematical functions can greatly contribute to the success of dental restorations [16, 17]. The current study analyzed the translucency and opalescence of CAD-CAM materials across a range of thicknesses (0.5mm to 2.0mm), which are commonly encountered in clinical restorations such as veneers, inlays, onlays, overlays, full crowns, and monolithic crowns [3, 12–15].

The findings of this study demonstrated that translucency and opalescence varied with different thicknesses. TP_00_ exhibited a continuous decline and curvilinear relationship with increasing thickness, consistent with previous studies [8, 14, 15, 38, 39]. While, the variations in opalescence (OP) were material-dependent, indicating differences among the materials. Thinner specimens exhibited greater differences in TP_00_ and OP between adjacent thicknesses compared to thicker specimens. We observed the highest average variations in TP_00_ (TP_00_ = 9.72) between 0.5mm and 1.0mm and the lowest (TP_00_ = 3.41) between 1.5mm and 2.0mm, aligning with findings by Bayindir et al. [38]. Similarly, Kang et al. found that TP decreased as the thickness of resin-based composites and glass–ceramics increased, particularly at lower thicknesses [14]. However, this observation may be attributed to the limitations of clinical spectrophotometer, as variations in accuracy have been reported between clinical spectrophotometer like Vita Easyshade V and laboratory spectrophotometer [40]. The observed range in OP was from 5.66 (0.5 mm LU) to 9.55 (0.5 mm VS), consistent with results reported by Shirani et al. [3]. However, none of the tested groups in this study exhibited opalescence comparable to that of enamel [9].

Studies on the correlation between translucency and thicknesses have been reported [16, 17, 41–44]. However, obtaining a precise relationship, particularly at low thicknesses, and reaching a conclusive mathematical formula have proven challenging due to significant variations among different studies. The study on monolithic zirconia stained with a coloring liquid by Kim et al. [41] reported a linear correlation between translucency and thickness. While an exponential relationship between translucency and thickness of glass ceramics and zirconia ceramics was described by Wang et al. [42] and Sulaiman et al. [43]. A logarithmic relationship of translucency and thickness was described by Brodbelt et al. [44], Erdelt et al. [16] and Schweiger et al. [17] for ceramic materials and zirconia, respectively. In this study, four regression curves (linear, exponential, logarithmic, and quadratic) of the tested materials were analyzed. The results revealed that the quadratic regression curves provided the best fit for TP_00_ in most materials, except for LS and CD, which exhibited a logarithmic regression curve. However, due to limitations in thickness variation, drawing a unified conclusion about the correlation was challenging.

Translucency change in CAD-CAM materials is particularly noticeable to patients and clinicians, as it is closely related to lightness, which is more perceptible to human eyes than hue or chroma [18]. Visual translucency difference thresholds have been widely used as a quality control tool to guide the selection of esthetic dental materials, assess clinical performance, standardize procedures, and interpret findings in clinical dentistry and dental research [19]. In our study, we observed average TP_00_ variations between adjacent thicknesses ranging from 3.64 (between 1.5mm and 2.0mm) to 9.85 (between 0.5mm and 1.0mm). Except for LU specimens, the variations of all groups exceeded the translucency acceptability threshold. These findings indicated that changes in translucency due to thickness were visually apparent. Therefore, careful attention should be given to the adjustment of restoration thickness, as variations of 0.5mm or more can lead to clinically noticeable and potentially unacceptable differences in translucency, particularly for restorations less than 2.0mm thick [14].

In the present study, the six tested CAD-CAM materials were evaluated based on their typical material types and common use in dentistry. Our findings revealed that translucency was primarily influenced by material type, whereas opalescence was more affected by thickness, contradicting the findings of Barizon et al. [39], who stated that translucency was primarily influenced by thickness. We observed significant differences in translucency and opalescence among the tested materials, with the VS specimens exhibiting significantly lower translucency and higher opalescence compared to the other groups. The LS and LU specimens showed the highest translucency and lowest opalescence, respectively. These results indicate that these materials cannot be used interchangeably in clinical situations, particularly for veneers, considering their differences in translucency and opalescence.

The influence of inner structures and compositions on translucency and opalescence has been reported in previous studies [45, 46]. Materials with higher mechanical properties tend to have lower translucency [47, 48]. Differences in light transmission characteristics among monolithic materials can be attributed to factors such as monomer and filler type and content, filler size, polymerization, defect distribution, porosity, and inorganic content [12, 46, 49]. The manufacturers of LS reported that this glass ceramic exhibits variations in translucency and opalescence due to the presence of large and small lithium meta-silicate crystals in the pre-crystallized state [45]. Differences in inorganic filler content may explain the variation in translucency between these materials [49]. Additionally, the presence of fillers with radio-opacifying properties can affect material translucency [12]. These factors contribute to the differences in translucency between resin-nano ceramic (LU) and polymer-infiltrated ceramic materials (VE). Zirconia-reinforced lithium silicate ceramics, such as SU and CD, have gained popularity in CAD-CAM systems due to their combination of esthetic properties from glass ceramics and strength from ZrO_2_ particles [50]. Consistent with previous studies, our results showed that CD, LS, and LU exhibited higher TP_00_ compared to other groups [14]. The nano size of ZrO_2_-SiO_2_ ceramic particles contributes to the translucency of the materials [51]. VS exhibited lower TP_00_ than CD and showed significantly higher opalescence, in line with the findings of Shirani et al. [3]. The sintering process after milling for VS may result in alterations in crystal size and structure, such as more compact interlocking of microstructures in crystals, thus leading to lower translucency and higher opalescence [52].

We also investigated the effect of different roughening treatments on translucency and opalescence [12]. Increasing surface roughness caused a reduction in TP_00_ and an increase in OP. As thickness decreased, the variations in TP_00_ and OP among the different roughening treatments increased. The influence of surface treatments on the translucency of restorative materials has been previously studied, demonstrating that roughness and topographical alterations affect light transmittance [29, 30]. This may be because light direction and incidence are altered when light transmits through a roughened surface, which may alter optical characteristics, especially material opacity [25, 29]. We observed that the difference in TP_00_ between the P300-grit roughened and the polished specimens in 0.5mm was 2.59 on average, exceeding the perceptible threshold for translucency and approaching the acceptability threshold [6]. The average TP_00_ difference decreased to 1.39 for 2.0mm thick specimens, still surpassing the perceptible threshold but falling below the acceptability threshold. These findings indicate that the translucency difference caused by roughening is perceptible and potentially clinically unacceptable. Moreover, the effect of roughening treatments on translucency and opalescence appeared to be material-specific. LS showed less variation in translucency and opalescence with different roughening treatments compared to other materials, while TE and VS exhibited the highest variation respectively. This phenomenon may be attributed to the greater hardness and dense internal molecular structure of lithium disilicate glass ceramics [45]. The same roughening treatments led to fewer changes in surface roughness, and, consequently, less variation in translucency and opalescence. Therefore, when selecting restorations, the surface condition of the material should be given equal consideration alongside translucency and opalescence. Posterior processing treatments, such as high-gloss polishing, play a crucial role in restoring the appearance of dental restorations based on the results of this study.

It is important to note some limitations of our study. Firstly, it should be noted that clinical spectrophotometers like Vita Easyshade V may not be as accurate as laboratory measuring instruments. Therefore, the results obtained from clinical spectrophotometers should be interpreted with caution, as the translucency and opalescence were not obtained using a laboratory spectrophotometer [40]. Secondly, the findings may not directly apply to clinical situations since the effects of underlying structures like abutments and luting agents were not considered. Thirdly, some materials used in our study can undergo glazing, which can influence their translucency and opalescence.

## Conclusions

Based on the limitations of our study, we draw the following conclusions:

1. The translucency and opalescence of CAD-CAM materials were significantly influenced by material type, thickness, and roughening treatment. Variations in thickness of 0.5 mm or greater may lead to unacceptable discrepancies in translucency.
2. CAD-CAM materials should be carefully chosen due to their different optical properties. LS and LU exhibited higher translucency, while SU and TE exhibited higher opalescence.
3. Roughening treatments had a significant influence on translucency and opalescence, which caused perceptible and even clinically unacceptable differences in translucency.

## Data Availability

All essential data is presented in the manuscript. The datasets and images are available from the corresponding author on reasonable request.
